# The *PIN1* family gene *PvPIN1* is involved in auxin-dependent root emergence and tillering in switchgrass

**DOI:** 10.1590/1678-4685-GMB-2014-0300

**Published:** 2016

**Authors:** Kaijie Xu, Fengli Sun, Yongfeng Wang, Lili Shi, Shudong Liu, Yajun Xi

**Affiliations:** 1 State Key Laboratory of Crop Stress Biology for Arid Areas, Northwest A&F University, Yangling, Shaanxi, China; 2 Institute of Cotton Research, Chinese Academy of Agricultural Sciences, Anyang, Henan, China; 3 Handan Academy of Agricultural Sciences, Handan, Hebei, China

**Keywords:** Auxin transporter, *PvPIN1* gene, TIBA treatment, tillering, switchgrass

## Abstract

Switchgrass (*Panicum virgatum* L.; family Poaceae) is a warm-season C4 perennial grass. Tillering plays an important role in determining the morphology of aboveground parts and the final biomass yield of switchgrass. Auxin distribution in plants can affect a variety of important growth and developmental processes, including the regulation of shoot and root branching, plant resistance and biological yield. Auxin transport and gradients in plants are mediated by influx and efflux carriers. *PvPIN1*, a switchgrass *PIN1*-like gene that is involved in regulating polar transport, is a putative auxin efflux carrier. Neighbor-joining analysis using sequences deposited in NCBI databases showed that the *PvPIN1*gene belongs to the *PIN1* family and is evolutionarily closer to the *Oryza sativa japonica* group. Tiller emergence and development was significantly promoted in plants subjected to*PvPIN1* RNA interference (RNAi), which yielded a phenotype similar to that of wild-type plants treated with the auxin transport inhibitor TIBA (2,3,5-triiodobenzoic acid). A transgenic approach that induced*PvPIN1* gene overexpression or suppression altered tiller number and the shoot/root ratio. These data suggest that *PvPIN1*plays an important role in auxin-dependent adventitious root emergence and tillering.

## Introduction

Switchgrass (*Panicum virgatum* L.; family Poaceae) is a perennial warm-season C4 grass. In recent years, switchgrass has received increasing attention as a highly versatile feedstock used for soil and water conservation, livestock feeding and bioethanol production (Casler, 2012; Parrish *et al*., 2012). However, the competition between switchgrass and crops for water and arable lands may exacerbate current food security challenges (Varvel *et al*., 2008). Therefore, maximizing the use of various non-irrigated marginal croplands and improving the resistance of switchgrass may be plausible approaches to address the above concern.

One of the practical strategies to enhance plant production and anti-stress abilities is to use molecules involved in various stages of plant growth. The plant phytohormone auxin regulates numerous developmental processes during plant growth, including growth direction, shoot and root branching, and differentiation of vascular tissue (Xu *et al*., 2005). Auxin also plays an important role in plant resistance and biological yield (Wang *et al*., 2007; Seo *et al*., 2009). Auxin is generally thought to be synthesized in young apical tissues and then transported down to the maturing stem and root via a polar transport system (Crozier *et al*., 2000). The cell-to-cell polar transport of auxin requires influx and efflux carrier proteins (Muday and DeLong, 2001) and is important for controlling critical developmental processes by reorienting appropriate growth in response to various environmental stimuli. For these reasons, there is considerable research into auxin transport.

The *PIN* family of genes is involved in regulating polar transport, and many effects of auxin can be blocked by inhibitors or mutations that prevent the polar transport of auxin (Rosen *et al*., 1999). The *PIN* family in*Arabidopsis* has eight members (*AtPIN1* to*AtPIN8*) (Chen *et al*., 2012). Each member exhibits a unique tissue-specific expression pattern and subcellular localization (Paponov *et al*., 2005). *PIN1* is an efflux-facilitating family member (Friml *et al*., 2003; Blilou *et al*., 2005) that encodes a putative auxin efflux carrier protein. PIN1-type proteins determine the direction of cell-to-cell auxin transport through their asymmetric subcellular localizations in the plasma membrane (Winiewska *et al*., 2006; Huang *et al*., 2010).

In *Arabidopsis*, a mutation in the *PIN1* gene (Okada *et al*., 1991) results in failure to establish endogenous auxin gradients, leading to the abnormal formation and development of shoots, roots, embryos and inflorescence (Vernoux *et al*., 2000; Benková *et al*., 2003; Reinhardt *et al*., 2003; Weijers and Jürgens, 2005). Homologs of the*PIN1* gene have also been identified in other plant species, including *Setaria italia* (GenBank: XM_004953823.1),*Zea* (Forestan and Varotto, 2010), *Oryza sativa* (Xu*et al*., 2005) and *Sorghum bicolor*(Paterson *et al*., 2005). In particular, overexpression of the rice*OsPIN1* gene (previously known as *REH1*) significantly increases the primary root length and lateral root number (Luschnig *et al*., 1998). The OsPIN1 protein is very important for determining tiller angle and number (Xu *et al*., 2005).

Like rice, switchgrass produces numerous adventitious roots that are essential for plant development, particularly for stress resistance. Use of the polar auxin transport inhibitor *N*-1-naphthylphthalamic acid (NPA) or 2,3,5-triiodobenzoic acid (TIBA) may block the development of rice root collars (the junction between the root and stem) but promote the initiation and growth of adventitious and lateral roots (Zhou *et al*., 2003). Auxin inhibits shoot branching (Chatfield *et al*., 2000) and the ectopic distribution of auxin could influence flower bud development at various stages. Auxin signaling may also play a key role in axillary bud development (Leyser, 2003).

Even though roles for *PIN1* in the formation and development of adventitious roots and tillers have been identified in many plant species, the function of *PIN1* in switchgrass has not been investigated. In this report, we examined the characteristics of the *PIN1* gene in switchgrass and its effects on the development of adventitious roots and tillers. The findings described here enhance our understanding of *PIN1* gene functions and may also stimulate basic genetic research into switchgrass.

## Materials and Methods

### Plant materials, explant sterilization and callus induction

A switchgrass cultivar 'Xiji 2' bred from the American 'Alamo' cultivar was used as the transgenic acceptor to induce callus formation. Mature caryopses were picked from a single plant and then surface-sterilized for callus induction. The resulting caryopses were further processed on two consecutive days (Xi *et al*., 2009). Initially, they were surface-sterilized with 70% ethanol for 1 min and 15% sodium hypochlorite for 75 min, rinsed 3–4 times with sterile water and left overnight at 4 °C. On the following day, they were sterilized with 15% sodium hypochlorite for 30 min and rinsed 3–4 times with sterile water. Next, sterilized caryopses were placed on induction medium (M5) composed of Murashige and Skoog (MS) basal medium supplemented with 22.6 μM 2,4-D (2,4-Dichlorophenoxy), 0.2 mg/L 6-BA (6-Benzylaminopurine), 1 mg/L picloram, and 1.5% sucrose and 1.5% mannitol (w/v) as the carbon sources; this medium was subsequently solidified with 0.7% agarose (w/v). The cultures were incubated in the dark at 25 °C for 4–6 weeks, after which calli were obtained and embryogenic calli were selected for transgenesis.

### Switchgrass *PvPIN1* gene cloning

Total RNA extraction (Invitrogen, Carlsbad, CA) from switchgrass shoots at the V3 stage (Moore *et al*., 1991) and cDNA synthesis (Takara, Shiga, Japan) were done as described in the manufacturers' protocols. Based on the transcriptomes of different tiller mutants, sequences similar to the *PIN1* gene were selected as differentially expressed genes and primers were designed for*PIN1* gene cloning. The open reading frame (ORF) of the*PIN1* gene was cloned from switchgrass. Polymerase chain reaction (PCR) amplification products were analyzed electrophoretically in 1% agarose/ethidium bromide gels. The fragments were subsequently cloned and inserted into T-easy vectors (Promega, Madison, WI), transformed in*Escherichia coli* DH5α and sent to Life Technologies (Carlsbad, CA) for sequencing.

### Construction of RNAi and expression vectors for*PvPIN1*

An RNA interference (RNAi) construct was generated to investigate the effects of loss of *PvPIN1* function. The pTCK303 vector (Wang *et al*., 2004) contains a chimeric hygromycin phosphotransferase (*HPT*) gene and a β-glucuronidase (*GUSa*) gene, both under control of the CaMV35S promoter. According to the pTCK303 vector structure, a 491-bp fragment from the ORF of *PvPIN1* was amplified using two sets of primers (dF1 and dR1); another fragment was similarly amplified using primers dF2 and dR2 (Table 1). The resulting DNA fragments were first cloned into T-easy vectors (Promega) for sequence verification by Life Technologies. After confirmation, the two vectors and the pTCK303 vector were digested using double digestion and recycled using a gel extraction kit (Tiangen Biotech, Beijing, China). The fragments were subsequently inserted separately into the pTCK303 vector using T4 ligase. The chimeric RNAi vector was transformed into DH5α for transgenesis.

**Table 1:** Sequences of the primers used in this work.

Primer name	Sequences (5'-3')	Enzyme sites
F	ATGATTACGGGGGCGGACTT	
R	TCACAACCCGAGCAGGATGT	
F1	CGGGATCCCGATGATTACGGGGGCGGACTT	*Bam*HI
R1	GACTAGTCTCACAACCCGAGCAGGATGT	*Spe*I
dF1	GACTAGTCGGCATCAACCGCTTCGTCGC	*Spe*I
dR1	CGAGCTCGTGTATCTTGCCGTCCTCCTT	*Sac*I
dF2	GGGGTACCCCGGCATCAACCGCTTCGTCGC	*Kpn*I
dR2	CGGGATCCCGTGTATCTTGCCGTCCTCCTT	*Bam*HI
HphF	GGAGCATATACGCCCGGAGT	
HphR	GTTTATCGGCACTTTGCATCG	
HphF1	TTCTACACAGCCATCGGTCCA	
HphR1	TTAGCCAGACGAGCGGGTTC	
qF	GAGTTCAGCTTCGGGAACAG	
qR	CTCGAAGTTCCACCTGAAGC	

To study *PvPIN1* gain of function, the full-length coding region of the *PvPINI* gene was amplified using the F1/R1 primer pairs with *Bam*HI and *Spe*I restriction sites, respectively. The resulting PCR product was cloned in the T-easy vector (Promega) and then inserted into the pTCK303 vector. Plasmid DNAs were extracted according to the manufacturer's protocol (Plasmid DNA extract kit, Tiangen Biotech).

### Particle bombardment and genetic transformation

A plasmid containing RNAi and *PvPIN1* gene expression vectors was extracted according to the manufacturer's protocol (Plasmid DNA extraction kit, Tiangen Biotech). Before particle bombardment, the embryogenic calli were placed in a 4-cm^2^ circle monolayer on MSH (Hypertonic MS) medium in a 9-cm dish containing MS medium basal medium with 22.6 μM 2,4-D and 0.4 M mannitol (Sigma, St. Louis, MO) followed by a 4–6 h osmotic treatment in the dark at 25 °C. Gold particles (1 μm, Bio-Rad, Hercules, CA) were soaked with the above-mentioned plasmid DNA (Vain *et al*,. 1993). For each bombardment shot, 1 μg of plasmid DNA and 250 μg of gold particles were used. The bombardment was done using a 1100 psi He inflow with a target distance of 9 cm. Thereafter, the bombarded calli were incubated on MSH medium overnight.

### Transgenic plant selection and regeneration

*PvPIN1* gene function was verified by selecting embryogenic calli and bombarding them with microprojectiles carrying pTCK303 vectors (containing the RNAi and overexpression vectors) with a gene gun (Bio-Rad). Initially, the bombarded calli were transferred to the recovery medium and 22.6 μM 2,4-D was added to the MS basal medium one week later. The recovered calli were selected on selection medium that contained hygromycin B (50 mg/L) at 25 °C in the dark for 4–5 weeks. The calli were cultured at 25 °C on regeneration medium containing kinetin (KT, 2 mg/L) to induce cell division and callus differentiation and development. An illumination intensity of 12,000 μmol/(m^2^.s) and a 16/8 h (light/dark) photoperiod was used until the calli reached bud differentiation. The seedlings were then transplanted into soil in containers for further root development on a 12 h photoperiod at 28/25 °C and 60% humidity in a phytotron.

### Molecular analyses of transgenic plants

The *PvPIN1* gene sequences were inserted between the two reporter genes and controlled by a UBi-1 promoter. The GUS reporter gene contains a catalase intron within the coding sequence to ensure eukaryote-specific expression. The seedlings were allowed to grow to the V3 stage and DNA samples from each plant were extracted using the 2x CTAB method. These DNA samples were screened using PCR with the Hyg and GUSa primers (Table 1) and *Taq* DNA polymerase (Fermentas, Burlington, ON, Canada). The expected product sizes were 612 bp for Hyg and 1,081 bp for GUSa. The components and conditions of the PCR were similar to those used for *PvPIN1* gene cloning, except that the templates used were the genomic DNA of regenerated seedlings and the extension time was 90 s. The resulting PCR products were analyzed by electrophoresis in 1% agarose/ethidium bromide gels (Xi *et al*., 2009).

For Southern blot hybridization analysis, genomic DNA was extracted from 250 mg of fresh young leaves of transgenic seedlings using a Plant Genomic DNA kit (Tiangen Biotech). The DNA content of the samples was quantified prior to digestion with the restriction enzyme *EcoRI* (Takara), which only digests the T-DNA region, and each sample was then hybridized with the hygromycin probe. Briefly, 12 μg of DNA from each sample was digested overnight and loaded onto each lane. The hybridization probe (hph) was labeled with digoxigenin (DIG, Roche, Basel, Switzerland) by PCR. The primers for the hybridization probe were HphF1 and HphR1, and the expected product size was 590 bp. Gel electrophoresis, DNA blotting and hybridization were done according to the manufacturer's instructions. The hybridization signals were detected using the chemiluminescent substrate CSPDStar (Roche).

Southern blotting was used to examine the insertional copy numbers of individual transgenic plants. To eliminate differences in the genetic background of the plants, ten separate transgenic lines were selected from each group (PvPIN1-Ri, CK (wild-type) and PvPIN1-OE) that had strong PCR bands and Southern blot results for phenotypic analysis. The old and young tillers and roots of these plants were dissected and genomic DNA was extracted. RNA quality and quantity were analyzed using an Agilent 2100 Bioanalyzer (Agilent Technologies, Santa Clara, CA) and gel electrophoresis. Meanwhile, the apical and basal shoots and roots of selected transgenic plants were dissected and processed for enzyme-linked immunosorbent assays (ELISAs) for endogenous auxin; the assays were done at the China Agricultural University (Yang *et al*., 2001). Phenotypic data for the three groups were collected and analyzed using Excel 2007 (Microsoft, Redmond, WA) and the figures were drawn with Origin 7.5 (Origin Lab, Northampton, MA).

### qRT-PCR Analysis

Quantitative reverse transcriptase (qRT)-PCR (Takara) was used to quantify*PvPIN1* expression in transgenic (containing RNAi and overexpressing *PvPIN1*) and wild-type plants. Total RNA was extracted from the young tillers of wild-type and transgenic switchgrasses with TriReagent (Invitrogen) and reverse transcribed for cDNA according to the manufacturer's protocols (Takara). SYBR Green (Takara) was used as the reporter dye. The qRT-PCR primers are listed in Table 1. The cycle thresholds were determined using a Bio-Rad CFX96 sequence detection system with a volume of 25 μL containing 12.5 μL of*Taq*Man Master Mix SYBR Premix ExTaq (Takara). The cycling conditions were as follows: 95 °C for 10 min, followed by 40 cycles of 95 °C for 15 s and 60 °C for 1 min. Two microliters of cDNA (80 ng) per sample was used for PCR amplification. The data were normalized using the level of switchgrass β-actin transcripts. The primers were 5'-CACTGGAATGGTCAAGGCTG-3' (forward primer) and 5'-CTCCATGTCATCCCAGTTG-3' (reverse primer) (Song *et al*., 2012).

### TIBA treatment

To determine the role of *PvPIN1* in auxin transport, TIBA was used to disrupt the polar transport of this hormone in the lateral buds and stem nodes. The top nodes at the E4 stage represent lateral bud formation and the stem nodes with lateral shoots were collected and cut into 3-cm pieces with the node in the middle. The nodal segments were surface sterilized with 70% ethanol for 1 min, followed by 15% NaClO (9% effective chlorinity) for 15 min, and then rinsed three times with sterile water. The resulting materials were then inoculated on MSB medium (MS medium supplemented with 3 mg/L 6-BA, 3% sucrose and solidified with 0.7% agar). The buds grew to 3–5 cm in length after exposure to light at 25 °C for 2–3 weeks. After bud removal, the nodes were further subcultured on MSB medium supplemented with 10 μM TIBA for two weeks (the TIBA solution was sterilized by filtration and added to the sterilized MSB medium).

## Results

### *PvPIN1* gene cloning and sequence analysis

**Figure 1:**
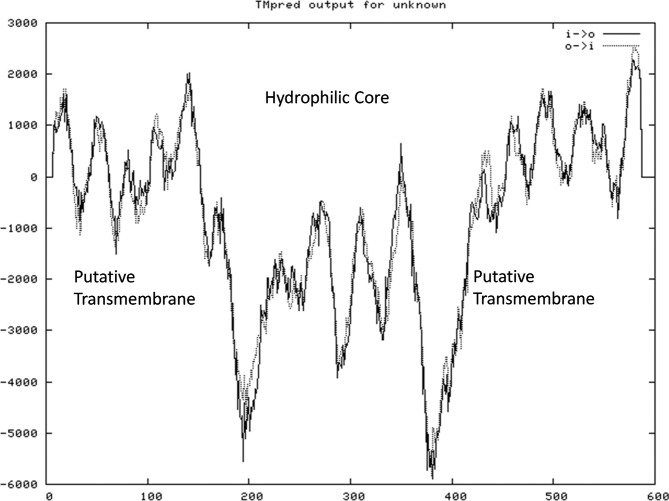
The membrane-spanning region and its orientation in the PvPIN1 protein as predicted by Tmpred.

**Figure 2:**
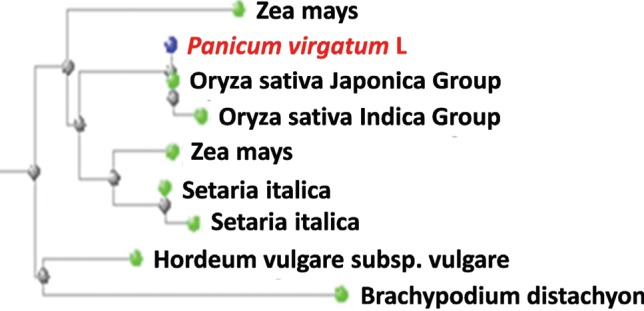
Phylogenetic analysis of PIN1 genes using the neighbor-joining method based on data collected from the NCBI database.

Analysis of the transcriptome results for switchgrass tillering mutants indicated that the *PIN1* gene was differentially expressed. Based on the sequence in the transcriptome database, the *PvPIN1* gene was amplified from the cDNA of switchgrass young tillers with the primer pairs F and R (Table 1). The ORF of the*PvPIN1* gene contained 1,788 nucleotides that encoded a protein with 596 amino acid residues and had a calculated molecular mass of 64.7 kDa. Based on the hydropathy and transmembrane motif analyses, the deduced amino acid sequence of PvPIN1 was similar to that of the OsPIN1 protein, which contains three significant motifs: a putative hydrophilic core and two putative transmembrane domains on either side (Figure 1) (Luschnig *et al*., 1998; Xu *et al*., 2005). The PvPIN1 protein showed 94% sequence homology with OsPIN1 (sequence information collected from the NCBI database) and neighbor-joining phylogeny showed that PvPIN1 was grouped with the *Oryza sativa japonica* clade (Figure 2). Based on the foregoing analyses, this homologous switchgrass gene was provisionally named the *PvPIN1* gene.

### Regeneration of transgenic switchgrass

**Figure 3:**
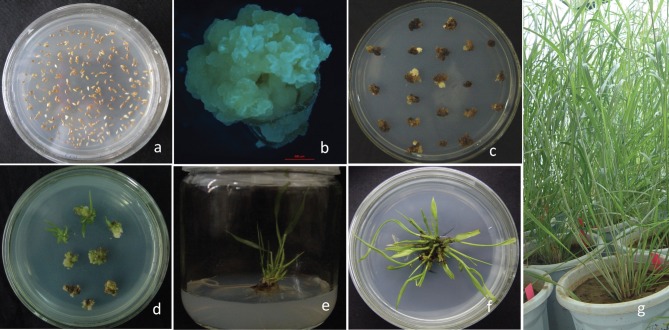
Transgenic switchgrass (*Panicum virgatum* L.). (A) Sterilized caryopses placed on induction medium (M5) to stimulate callus growth, (B) Embryogenic calli grown on osmotic medium, (C) Bombarded calli were selected on selection medium, (D) Surviving calli were regenerated on regeneration medium, (E, F) Regenerated seedlings, and (G) Seedlings in a phytotron.

**Figure 4:**
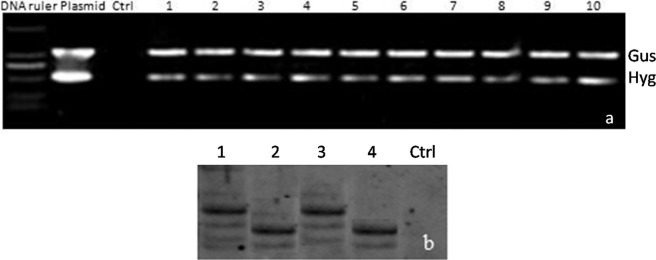
Molecular characterization of transgenic switchgrass plants. (A) Polymerase chain reaction (PCR) analysis of DNA samples from regenerated switchgrass plants with *Gus* and *Hyg*primers. Marker – 2000-bp DNA molecular markers. Plasmid – pTCK303 vector serving as positive control. Ctrl (Control check) – Wild-type plant serving as a negative control. Lanes 1–13 represent PCR products from individual transgenic plants: 1–4 represent PvPIN1-Ri (RNAi of the*PvPIN1* gene), 5–8 represent PvPIN1-OE (overexpression of the *PvPIN1* gene), 9 represents asexually reproduced seedlings of PvPIN1-Ri, and 10 represents asexually reproduced seedlings of PvPIN1-OE. (B) Southern blot hybridization analysis of the regenerated plants. The blot was hybridized with a DIG-labeled PCR product of the hygromycin gene. Lane 1 – PvPIN1-OE, 2 – PvPIN1-Ri, 3 and 4 – Asexually propagated plants.

After selection on hygromycin and regeneration of transgenic calli, the transgenic seedlings (Figure 3) were obtained and planted in a greenhouse. When the seedlings reached the V3 stage, DNA from individual plants was extracted using the cetyltrimethylammonium bromide (CTAB) method and the DNA samples were screened by PCR using the Hph (621 bp) and GUSa primers (1081 bp). The results showed that 61.9% of the T1 plants were positive for Hph and GUSa gene insertions (Figure 4A). Forty-five seedlings contained the overexpressed vector and 39 seedlings contained the RNAi vector. To exclude false positives produced during PCR amplification, true transgenics were identified based on clear, strong PCR bands (Xi *et al*., 2009).

To further eliminate false positives, Southern blot hybridization analysis was used to screen the putatively positive transgenic plants. The restriction enzyme*EcoRI*, which only digests once in the T-DNA region, was used to digest the genomic samples that were then hybridized with the hygromycin probe. Hybridization signals corresponding to the high-molecular weight bands and fragments of different molecular weights were observed (Figure 4B). Moreover, the asexually propagated seedlings had similar PCR and hybridization bands, and the *PvPIN1* gene was actually inserted in the genome and inherited in subsequent generations.

### *PvPIN1* gene expression pattern

**Figure 5:**
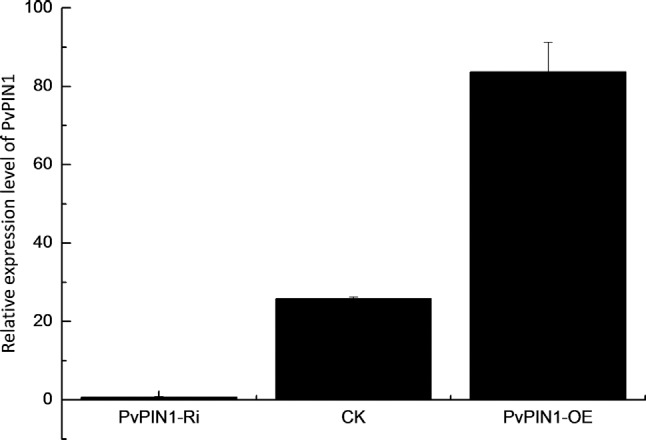
*PvPIN1* gene expression in CK (wild-type) and transgenic switchgrasses. The columns are the mean ± SD (n = 10 from separate transgenic lines).

To evaluate *PvPIN1* gene function during switchgrass development, vegetative tillers were collected from transgenic seedlings and subjected to qRT-PCR. *PvPIN1* gene transcript levels were high (2–4-fold increases) in the overexpressed lines and very low (undetectable) in the RNAi lines (Figure 5). This result demonstrates that vector construction and expression successfully generated transgenic plants.

**Figure 6:**
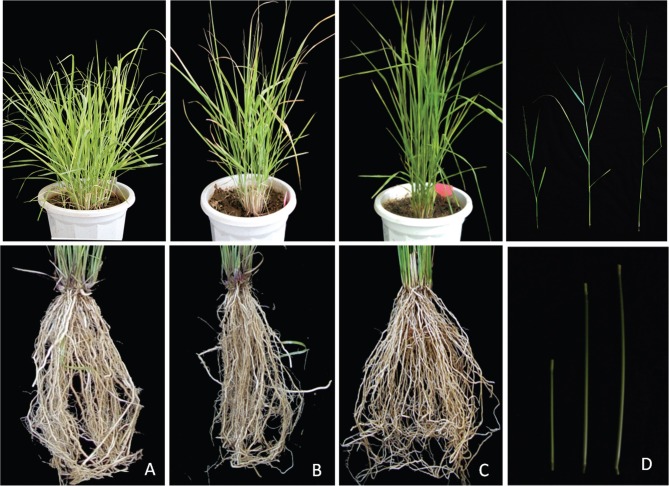
IAA (Indole-3-acetic acid) concentration (ng/g&middot;Fresh weight). The columns represent the mean ± SD (n = 3).

Plant hormone levels were measured by ELISA at the China Agricultural University in Beijing. The IAA (Indole Acetic Acid) levels of wild-type switchgrass and plants with RNAi and *PvPIN1* overexpression were analyzed. The concentrations of auxin in plants with *PvPIN1* gene RNAi were increased in the shoot apical meristem and higher than in wild-type plants, whereas the auxin concentration in the stem base was reduced and lower than in wild-type plants. In the overexpression lines, auxin levels were markedly increased in the stem base and significantly higher than in the control and in plants underexpressing *PvPIN1*. However, in the shoot apical meristem, the auxin levels were slightly elevated rather than reduced (Figure 6).

**Figure 7:**
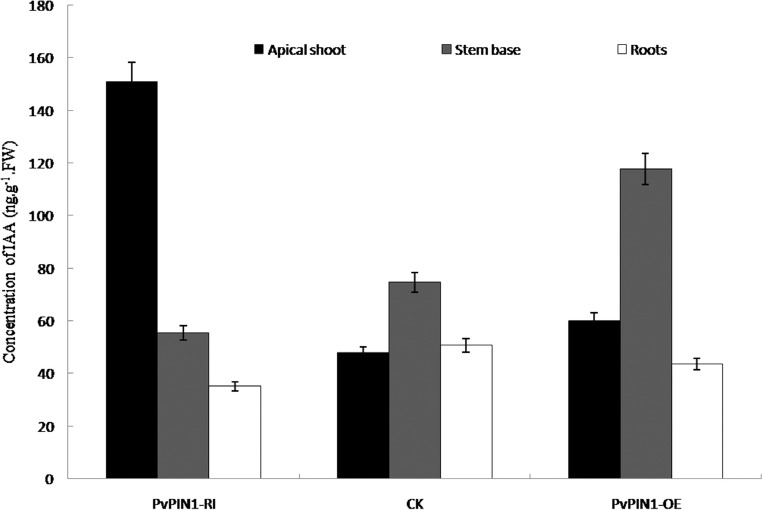
Phenotype differences between transgenic and wild-type (CK) switchgrass plants. Tiller number (A), tiller angle (B), root number (C) and seedling biomass yield (D) in transgenic and wild-type switchgrasses with different levels of *PvPIN1* gene expression. The columns represent the mean ± SD (n = 10).

**Figure 8:**
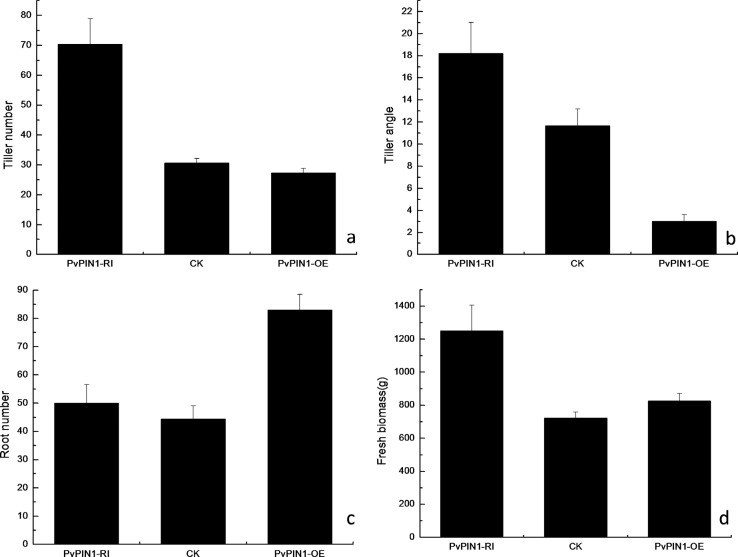
Transgenic switchgrass phenotypes. (A) PvPNI1-Ri, (B) CK(wild-type), (C) PvPIN1-OE, (D) PvPNI1-Ri (left), CK (wild-type, middle), PvPIN1-OE (right).

The effect of *PvPIN1* expression on switchgrass tillers was determined by measuring the tiller number (Figure 7A), tiller angle (Figure 7B), root number (Figure 7C) and fresh biomass per plant (Figure 7D). In contrast to wild-type plants, *PvPIN1* overexpression reduced the tiller number and angle but increased the root number and the fresh biomass per plant. The tiller angles of plants with *PvPIN1* RNAi were nearly 1.5 times higher than those in wild-type plants and six times higher than the overexpressing *PvPIN1* plants. In contrast, plants underexpressing *PvPIN1* showed marked increases in tiller and angle and fresh biomass per plant (Figure 8).

**Figure 9:**
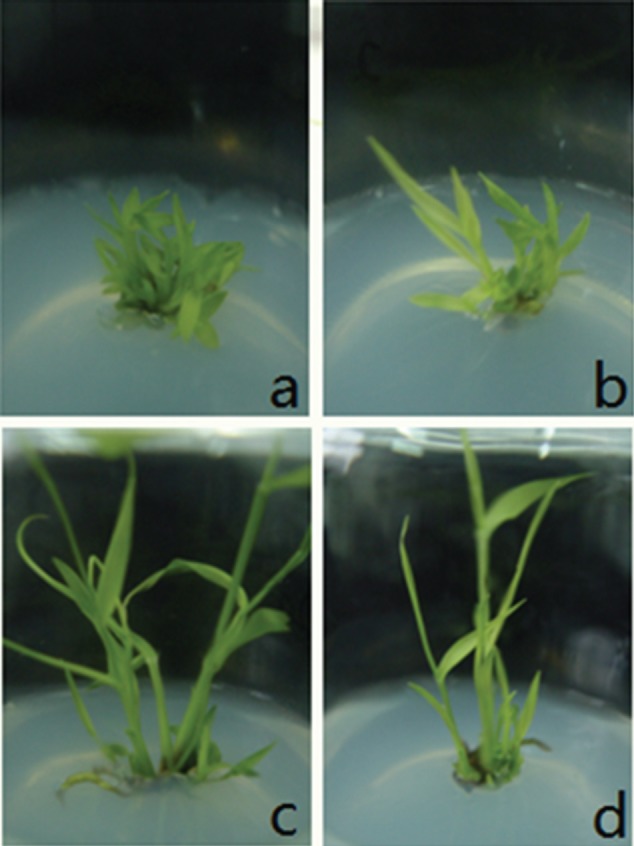
Phenotypic appearance of (A) TIBA-treated plants, (B) PvPNI1-Ri plants, (C) CK (wild-type) plants and (D) PvPIN1-OE plants.

Suppression of *PvPIN1* gene expression also promoted lateral bud outgrowth. Consequently, the number of tillers in *PvPIN1* RNAi transgenic plants was significantly greater than in control plants. When seedlings overexpressing *PvPIN1* were planted on MSB medium with and without TIBA, bud multiplication was more frequent on the former (Figure 9). Treatment with TIBA had a similar effect on wild-type switchgrass, suggesting that underexpression of the*PvPIN1* gene could affect auxin transport.

## Discussion

Tillering plays an important role in determining crop yield. Seedling tillering is controlled by heredity, photo-period, soil moisture, light intensity, temperature, mineral nutrition and pruning treatment to simultaneously alter bud and stem development (Moore *et al*., 1991). Switchgrass is an energy crop with strong tillering capacity. However, compared with other crops, there have been few studies of switchgrass tillers, particularly with regard to the identification of genes that control the tillering process.

The *PvPIN1* gene was one of the most highly differentially expressed genes in a pair of transcriptomes from switchgrass tiller mutants that showed significantly lower tiller numbers. *PIN1* genes in other plants have been reported to encode polar auxin transport proteins and affect tiller development (Gälweiler *et al*., 1998;Xu *et al*., 2005). In the present study, specific primers were designed by comparing the coding sequence of the *PvPIN1* gene in switchgrass with those of other*PIN1* genes. To predict protein function, the*PvPIN1* gene was translated into a 596-amino acid protein. Phylogenetic analysis revealed that the putative PvPIN1 protein belonged to the highly conserved PIN1 protein family, the members of which contain a hydrophilic region interconnecting two blocks of transmembrane segments (Palme and Gälweiler, 1999). This finding indicated that the gene of interest was a *PIN* family gene that may participate in auxin polar transport.

Tillering in switchgrass is one of the most important processes that directly determine biomass yield (Muir *et al*., 2001) and has been the principal trait for improving switchgrass breeding programs in past years (Adler*et al*., 2006). The phenotypes of transgenic switchgrass plants overexpressing the *PvPIN1* gene were similar to those of rice overexpressing *OsPIN1* (Xu *et al*., 2005). Specifically, they exhibited few tillers, small tiller angles and more roots. Conversely, reducing*PvPIN1* expression increased the tiller number, angle and fresh biomass per plant. In addition, treatment with TIBA, which strongly inhibits auxin efflux (Geldner *et al*., 2001), effectively blocked the polar transport of auxin and resulted in the same phenotypes as that of plants with reduced *PvPIN1* gene expression. These results indicated that the *PvPIN1* gene might play a role in polar auxin transport. Because the *PvPIN1* gene was predicted to encode a protein for auxin polar transport, differences in the*PvPIN1* gene expression patterns could lead to different auxin distributions that might in turn cause variations in plant phenotypes.

In plants treated with RNAi against the *PvPIN1* gene, the auxin concentration increased in the shoot apical meristem and decreased in the stem base; this inconsistent pattern led to the outgrowth of more tillers and a larger tillering angle. Conversely, in lines overexpressing *PvPIN1*, increased auxin levels in the base of the stem resulted in fewer tillers and smaller tillering angles (Figure 7 and Figure 8). Thus, overexpression of*PvPIN1* reduced the tiller number and angle and increased the root number, with both of these outcomes being beneficial to plant adaptability and increased biomass (Li *et al*., 1999). However, in the roots of plants with *PvPIN1*underexpression, the auxin levels were decreased but plant primary root length while lateral root numbers were not significantly affected. Studies of*Arabidopsis* have shown that *AtPIN1* plays an essential role in the initiation and development of lateral root primordia (Benková *et al*., 2003). A similar study reported that underexpression of *OsPIN1* by RNAi in rice reduced the number of adventitious roots (Geldner *et al*., 2001).

To further elucidate the reason underlying these differences, root auxin levels were measured and found not to be significantly different between the two transgenic switchgrass plants. One possible explanation for this is that there are other*PIN1* genes in switchgrass, as has been reported in rice (Wang *et al*., 2009). These*PIN1* genes may be functionally complementary in controlling the development of later roots. *PIN1* gene functions in switchgrass need to be studied further. It is thought that five *PIN* genes control auxin distribution to regulate cell division and cell expansion in the primary root (Blilou *et al*., 2005). However, another report concluded that little auxin is synthesized in the root; rather, most auxin in the root is obtained from the ground by acropetal translocation (Swarup *et al*., 2001). This mode of nonpolar transport of auxin in the phloem occurs in many plants (Goldsmith *et al*., 1974; Friml, 2003). The use of other transportation mechanisms may help to maintain the auxin gradient and regulate plant development.

In conclusion, we found that switchgrass *PvPIN1* acts as a polar auxin transporter based on the predicted protein structure, transgenic switchgrass phenotypes and TIBA treatment results. Switchgrass is an allogamous crop that is strongly self-incompatible (Talbert *et al*., 1983) and its different genetic backgrounds make the verification of gene function challenging. Fortunately, we obtained a large number of transgenic plants and were able to perform statistical analyses to eliminate inter-individual differences in our experiments. To our knowledge, this is the first report to describe the sequence of the PIN1 gene and its functions in switchgrass. The finding that *PvPIN1* is involved in auxin-dependent tillering emergence and adventitious root development provides new insight into the function of the *PIN1* family and should encourage the study of tillering in switchgrass.
